# Comparative Study of AlSi10Mg and 304 Stainless-Steel Fillers in PA12 Composites Manufactured Using Injection Moulding Process for Liners and Sleeve-Based Applications: Microstructure, Mechanical Properties, Thermal Stability, and Wear Behaviour

**DOI:** 10.3390/polym17202785

**Published:** 2025-10-17

**Authors:** Nabeel Maqsood, Bilal Islam, Karolis Stravinskas, Oleksandr Kapustynskyi, Romuald Petkevič, Alireza Shahidi, Genrik Mordas

**Affiliations:** 1 3D Technologies and Robotics Laboratory, Department of Laser Technologies, Center for Physical Sciences and Technology, Savanoriu Ave. 231, LT-02300 Vilnius, Lithuania; 2 Department of Mechanics and Material Engineering, Vilnius Gediminas Technical University, Plytines g. 25, LT-10105 Vilnius, Lithuania

**Keywords:** polymer composites, metal additive fillers, injection moulding, process optimization, mechanical properties

## Abstract

This study presents a comparative evaluation of injection-moulded PA12 composites reinforced with AlSi10Mg and 304 SS fillers, with emphasis on microstructure–property correlations linking powder morphology, mechanical performance, thermal stability, and tribological behaviour. Powder characterization revealed distinct morphologies—fine spherical AlSi10Mg particles (D50 ≈ 32 µm) dispersed uniformly in the matrix—while SS particles (D50 ≈ 245 µm) tended to agglomerate, leading to interfacial voids. Tensile testing showed that the elastic modulus of neat PA12 (0.95 GPa) increased by 20% and 28% with 20 wt% AlSi10Mg and SS, respectively. However, tensile strength decreased from 35.04 MPa (PA12) to 32.18 MPa (20 wt% AlSi10Mg) and 31.03 MPa (20 wt% 304 SS), consistent with stress concentrations around particle clusters. Hardness values remained nearly unchanged at 96–98 Shore D across all composites. Thermal analysis indicated that AlSi10Mg promoted crystallization, increasing crystallinity from 31% (PA12) to 34% and raising Tm by 2 °C. In contrast, 304 SS reduced crystallinity to 28% but significantly improved thermal stability, shifting Tonset from 405 °C (PA12) to 426 °C at 20 wt%. Tribological tests demonstrated substantial improvements: the coefficient of friction decreased from 0.42 (PA12) to 0.34 (AlSi10Mg) and 0.29 (304 SS), while wear rates dropped by 40% and 55%, respectively. SEM confirmed smoother worn surfaces in AlSi10Mg composites and abrasive grooves in 304 SS composites. The findings show that AlSi10Mg is advantageous for smoother surfaces and improved crystallinity, while SS enhances stiffness, wear resistance, and thermal endurance, providing design guidelines for PA12 composites in aerospace, automotive, and engineering applications.

## 1. Introduction

The growing demand for high-performance materials in various industrial sectors has led to significant advancements in composite material manufacturing. The development of advanced materials with tailored properties has been a cornerstone of modern engineering and manufacturing. Among these, polymer-based composites reinforced with carbon and metal particles have garnered considerable interest due to their superior mechanical, thermal, and electrical properties. These composites offer a unique combination of lightweight characteristics, enhanced strength, conductivity, and resistance to environmental degradation, making them ideal for structural and functional applications [1,2,3]. These composites are increasingly being used in industries such as aerospace, automotive, electronics, and biomedical engineering, where performance and efficiency are paramount [4,5].

Polymers, as a base material, provide flexibility, ease of processing, and corrosion resistance, while the incorporation of carbon-based fillers (such as carbon fibers, graphene, or carbon nanotubes) and metal particles (such as copper, aluminum, or nickel) enhances mechanical, thermal, and electrical properties [6,7,8]. The synergy between these components allows for the creation of multifunctional materials that can meet the demanding requirements of modern applications. However, the successful production of these composites requires a deep understanding of the interactions between the polymer matrix and the fillers, as well as the optimization of processing parameters to achieve uniform dispersion and strong interfacial bonding [9,10,11].

Carbon-based fillers such as carbon fibers (CF), carbon nanotubes (CNTs), and graphene are widely employed in polymer matrices due to their exceptional mechanical strength, electrical conductivity, and thermal stability. Similarly, metal fillers such as aluminum (Al), copper (Cu), and stainless steel (SS) are incorporated to enhance the electrical and thermal conductivity of polymer composites, making them suitable for applications in electromagnetic shielding, heat dissipation, and structural reinforcement. However, achieving uniform dispersion of fillers and optimizing the interfacial bonding between the polymer matrix and reinforcement remain critical challenges [7,12,13,14].

One of the most efficient and widely used manufacturing techniques for producing polymer composites is the injection moulding process. This process allows for high precision, cost-effective mass production, and excellent repeatability, making it particularly suitable for fabricating polymer-based composites with complex geometries [12,15,16]. The injection moulding process involves the melting of polymer pellets mixed with reinforcement materials (such as carbon or metal particles) and injecting the molten mixture into a mould cavity under high pressure [17,18,19]. Upon cooling and solidification, the final composite product exhibits enhanced properties based on the type and dispersion of the reinforcing materials. Therefore, a systematic approach to characterizing the produced composites is essential to ensure their performance and reliability.

Several researchers have explored the incorporation of carbon-based fillers into polymer matrices to improve the mechanical, electrical, and thermal performance of the resulting composites. Sorayani Bafqi et al. [20] examined the design and fabrication of high-performance thermoplastic composites reinforced with carbon and metal fillers. Their work emphasized the role of extrusion and injection moulding techniques in achieving enhanced mechanical and thermal stability. The study revealed that incorporating metal fillers significantly improved thermal conductivity while maintaining structural integrity. Meleshin et al. [21] conducted extensive testing of polymer composite materials and evaluated their mechanical properties. They utilized various polymer and filler combinations to determine their effects on strength, flexibility, and thermal resistance. Their findings highlighted that uniform dispersion of fillers was critical in optimizing performance, and injection moulding parameters played a significant role in achieving this uniformity. Głogowska et al. [22] analyzed the impact of metal powder fillers in hybrid moulded polypropylene composites. Their findings revealed that while metal fillers enhance tensile and compressive strength, they may also increase material brittleness. Fu et al. [12] provided a comprehensive overview of how injection moulding technology affects the mechanical performance of polymer composites and explored the correlation between processing parameters and final composite properties.

Despite the extensive research on polymer-based carbon and metal composites, several challenges remain, including optimizing filler dispersion, enhancing interfacial bonding, and refining processing conditions to achieve superior mechanical and thermal properties. The novelty of this research lies in the systematic investigation of the injection moulding parameters and their influence on the structural and functional properties of carbon-metal polymer composites. By incorporating advanced characterization techniques, this study aims to bridge the gap between experimental findings and industrial applications.

The present study introduces a comprehensive investigation into the fabrication and characterization of PA12-based composites reinforced with AlSi10Mg and 304 SS powders using the injection moulding process. Unlike prior research that primarily focused on single-filler systems, this work explores the synergistic potential of combining dual metallic fillers with PA12 to achieve an optimized balance of mechanical strength, wear resistance, and thermal performance. The novelty lies in establishing a clear microstructure–property–performance relationship by correlating injection moulding parameters, filler dispersion, and interfacial bonding with the resulting mechanical, hardness, surface, and tribological behaviors of the composites. Advanced characterization techniques, including SEM-EDS, surface roughness analysis, tensile testing, and tribological evaluation, are employed to provide in-depth insights into filler distribution and its impact on composite performance.

The key objectives of this research are to fabricate PA12 composites reinforced with AlSi10Mg and 304 SS powders using optimized injection moulding conditions, to systematically characterize their microstructural features and mechanical properties, and to assess the influence of filler type and loading on their functional performance. Additionally, this study aims to compare the experimental results with the existing literature to highlight the unique benefits of dual metallic reinforcement in PA12 and to establish guidelines for scaling up injection-moulded PA12–metal composites for industrial applications. By addressing critical gaps in understanding filler–matrix interactions and processing optimization, this work offers a pathway for the development of high-performance polymer–metal composites suitable for demanding engineering environments.

## 2. Materials and Methods

### 2.1. Material Grades Selection of Polymers and Additives

In this study, PA12 was selected as the primary polymer matrix owing to its excellent balance of mechanical strength, chemical resistance, low moisture absorption, and dimensional stability, which make it ideal for high-performance engineering applications. Commercially available PA12 powder (EOS GmbH, Krailling, Germany) was used due to its consistent quality and suitability for thermoplastic processing via injection moulding.

For reinforcement, two metallic fillers were incorporated: AlSi10Mg aluminum alloy powder and 304 SS powder. AlSi10Mg powder (LPW Technology Ltd., London, UK) was chosen for its low density, high thermal conductivity, and excellent corrosion resistance. The second reinforcement, 304 SS powder (LPW Technology Ltd.), was selected due to its high tensile strength, wear resistance, and ability to improve surface durability in polymer–metal composites.

### 2.2. Particle Size Distribution Evaluation

The particle size distribution (PSD) of PA12, AlSi10Mg, and 304 SS powders was measured using a laser diffraction particle size analyzer (Mastersizer 3000, Malvern Instruments Ltd., Malvern, UK) equipped with a dry dispersion unit. Prior to testing, the powders were dried at 80 °C for 12 h under vacuum to remove absorbed moisture and to prevent agglomeration during measurement. Each powder was fed into the dispersion unit under a controlled air pressure of 2 bar to ensure de-agglomeration without inducing particle breakage. Measurements were performed in triplicate, with each run consisting of 10,000 scattering events to ensure statistical reliability. The PSD was expressed in terms of the volume-based percentiles—D10, D50, and D90—corresponding to the particle diameters at which 10%, 50%, and 90% of the cumulative volume distribution is below that size, respectively.

### 2.3. Injection Moulding Process for the Fabrication of Polymer and Composites Parts

Prior to processing, all materials were dried in a vacuum oven at 80 °C for 8 h to remove moisture and prevent hydrolysis during injection moulding. The metallic powders (AlSi10Mg and 304 SS) were incorporated into PA12 at different weight fractions (10 wt.% and 20 wt.%) to study the effect of filler loading on the microstructural, mechanical, and tribological properties of the composites. The filler contents were selected based on prior studies indicating effective reinforcement within this range without inducing excessive viscosity or processing challenges.

The fabrication of PA12-based composites reinforced with AlSi10Mg and 304 SS powders was systematically designed to ensure uniform filler dispersion and compatibility with injection moulding. PA12 powder was used as the base matrix material instead of granules to facilitate better mixing and uniformity with metallic fillers. PA12 powder was mechanically premixed with AlSi10Mg and 304 SS powders at filler loadings of 10 wt.% and 20 wt.% using a tumble mixer operating at 60 rpm for 30 min to achieve preliminary homogeneity and reduce the risk of filler agglomeration.

**Figure 1:**
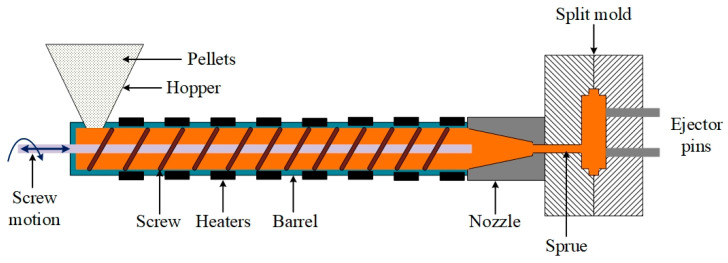
Schematic of the injection moulding process used to fabricate PA12 and metal-filled composites.

The premixed powders were then compounded using a injection unit (Desktop Injection Molding Machine BWMINI-15T, Taizhou, China) with a screw diameter of 18 mm and a length-to-diameter ratio (L/D) of 25:1. The barrel temperature profile was maintained between 190 °C at the feed zone and 230 °C at the die zone to achieve complete melting of PA12 while preserving the structural integrity of the metallic fillers. The screw speed was set to 80 rpm with a feed rate of 2 kg/h, and a vacuum vent was used to remove entrapped air and volatiles, ensuring void-free compounding. The extrudate was collected and cooled under controlled ambient conditions and subsequently broken down into smaller particles suitable for direct feeding into the injection moulding unit. A cooling time of 25–30 s was implemented to stabilize the moulded components and minimize internal stresses. Figure 1 illustrates the schematic of the injection moulding process used to fabricate metal-filled polymer composites.

### 2.4. Microstructural Analysis (SEM and EDS)

The morphology and distribution of metallic fillers within the PA12 matrix were examined using Scanning Electron Microscopy (SEM) integrated with Energy Dispersive X-ray Spectroscopy (EDS) (Axia™ ChemiSEM, Thermo Fisher Scientific, Waltham, MA, USA). SEM imaging was used to assess filler dispersion, matrix-filler interfacial adhesion, and the presence of voids or agglomerations. Additionally, EDS was performed to confirm the elemental composition of the AlSi10Mg and SS fillers and to verify their uniform distribution within the matrix.

### 2.5. Surface Roughness Analysis and 3D Surface Imaging

Surface roughness was measured using a non-contact CNC Vision Measuring System (Mitutoyo Quick Vision Hyper, Tokyo, Japan) with Mitutoyo QVPAK version 14.1 and Mitutoyo MCubeMap Ultimate V9 software version in accordance with ISO 21920 standards [23]. For each region, a scanning area of 1 mm × 1 mm was analyzed. Three measurements were taken on each specimen, and the arithmetic mean roughness (*Ra*) was reported. This analysis provided insights into the influence of metallic fillers and injection moulding on the surface finish and its potential implications for tribological behavior.

### 2.6. Tensile Testing

**Figure 2:**
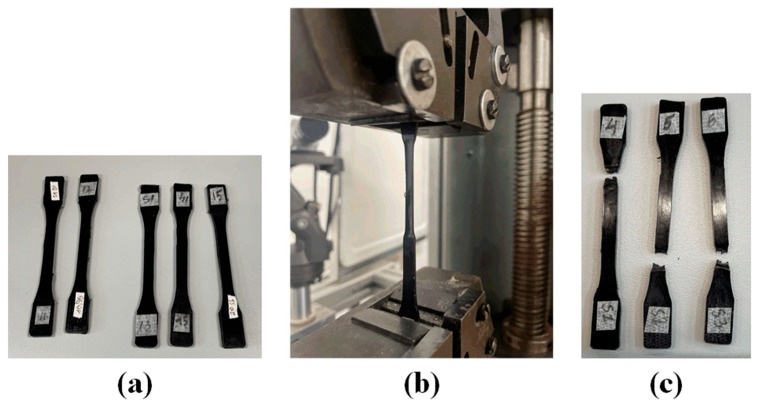
PA12 metal filled composite: (**a**) injection-moulded tensile samples; (**b**) tensile testing; (**c**) fractured samples after tensile testing.

The tensile properties of the polymer and composites were evaluated according to ISO 527-1 standard [24] using universal tensile testing instrument, 2055 P-0.5 (Tochpribor, Ivanovo, Russia), equipped with LabVIEW 2020 software with a 10 kN S-type tension load cell and a 3542 extensometer weas used for the measurements. Testing was carried out at a crosshead speed of 1 mm/min under ambient conditions (22 ± 2 °C, 50 ± 5% RH). Dogbone specimens were prepared with nominal dimensions of 105 mm × 8 mm × 3 mm (length × width × thickness). Key parameters measured included tensile strength and tensile modulus, which were compared across different filler loadings to assess reinforcement effectiveness. Figure 2 shows injection-moulded PA12 metal filled composite.

### 2.7. Hardness Testing

Shore D hardness was measured according to ISO 868 [25] using a calibrated durometer (ZwickRoell 3114, Ulm, Germany) mounted on a stand with a Type-D indenter. Rectangular plaques (≈80 × 10 × 4 mm^3^) were conditioned at 23 ± 2 °C and 50 ± 10% RH for at least 48 h before testing. Indentations were performed on as-moulded surfaces, avoiding edges, flow marks, and gate areas, with each indent separated by at least 10 mm. The indenter was applied perpendicularly without impact, and hardness values were recorded after a 15 s dwell time, as specified by the standard. For each formulation, five specimens were tested with 5–10 indents per specimen, and results are reported as mean ± standard deviation.

### 2.8. Tribological (Wear) Testing

The tribological behavior of the composites was assessed using a ball-on-disc wear test configuration following ASTM G99 standards [26]. A hardened steel ball (6 mm diameter) was used as the counter face under a normal load of 10 N, sliding distance of 1000 m, linear speed of 0.1 m·s^−1^, and ambient temperature (23 ± 2 °C, 40–50% RH). The wear rate was determined by measuring the volume loss using a 3D optical profilometer (Alicona InfiniteFocus, Lincoln, NE, USA). The coefficient of friction (COF) was recorded in real-time to evaluate the effect of filler addition on the tribological response.

### 2.9. Differential Scanning Calorimetry (DSC)

Thermal transitions of neat PA12 and its composites were measured by differential scanning calorimetry (DSC) using a TA Instruments DSC Q2000 (New Castle, DE, USA). Samples (~8–10 mg) were sealed in aluminum pans. The heating–cooling–reheating protocol was performed under nitrogen flow (50 mL·min^−1^) as first heating: 25 °C → 250 °C at 10 °C·min^−1^ (to erase thermal history), cooling: 250 °C → 25 °C at 10 °C·min^−1^ and second heating: 25 °C → 250 °C at 10 °C·min^−1^.

The glass transition (*Tg*) was determined from the midpoint of the heat capacity step during the second heating. The melting temperature (Tm) was taken from the endothermic peak maximum, and the crystallinity ($X_{c}$) was calculated using:

(1) $$X_{c}(\%)\;=\;\frac{\Delta H_{m}}{\Delta H_{m}^{0}.\;(1-w_{f})}\;\times \;100$$

where $\Delta H_{m}$ is the measured melting enthalpy, $\Delta H_{m}^{0}\;$ = 209 J·g^−1^ is the enthalpy of 100% crystalline PA12, and w_f_ is the weight fraction of filler.

### 2.10. Thermogravimetric Analysis (TGA)

Thermal stability was evaluated by thermogravimetric analysis (TGA) using a TA Instruments TGA Q500 (New Castle, DE, USA). Approximately 10 mg of each sample was heated from 30 °C to 800 °C at a rate of 10 °C·min^−1^ under nitrogen (50 mL·min^−1^). Additional oxidative stability tests were carried out in air atmosphere at the same heating rate. The following parameters were extracted: *Tonset*: temperature at 5% mass los, *Tmax*: temperature at maximum degradation rate (from DTG curves) and *Residue*: mass fraction remaining at 800 °C.

### 2.11. Design of Experiments (DoE)

The experimental design was clarified as a full-factorial study to quantify filler type and loading affect PA12 microstructure, mechanics, thermal behavior, and tribology. Specifically, two fillers (AlSi10Mg and 304 SS) across three loadings (0, 10, and 20 wt%) were tested, analyzing results both categorically and versus volume fraction (ϕ). Replication was predefined per cell: tensile (*n* = 5; extensometer), hardness (Shore D, *n* = 10 across ≥5 specimens), tribology (ball-on-disc, *n* = 3; steady-state COF and specific wear from 3D profilometry), roughness (*n* = 5 surfaces with 3 readings each; Sa or Ra consistently), DSC/TGA (*n* = 3), density (Archimedes, *n* = 3), and SEM stereology (≥6 fields-of-view at two magnifications). Molding and testing orders were randomized and operator/instrument sessions balanced across blocks. Primary responses were elastic modulus, tensile strength, COF, wear rate, crystallinity (from second heating), TGA onset/Tmax, porosity, and dispersion indices; secondary responses included hardness, roughness, density, and DSC metrics (Tm, ΔHm, Cp at 25 °C). Specimens with obvious molding defects were pre-screened; tensile curves were excluded only for grip slippage or fracture outside gauge, and all exclusions are reported. Figures present means ± SD and model bands with 95% CIs, and the chosen replication affords 80% power to detect 10–12% changes in modulus under typical variance.

## 3. Theoretical Approximations

### 3.1. Mathematical Relationship for Ultimate Tensile Strength

Ultimate tensile strength (σ_UTS_) can be interrelated with concentration of filler (X_conc_) mathematically by assuming change in concentration of fillers i.e., Al or SS. Moreover, this change in filler concentration will affect the σ_UTS_ values by considering a constant variation. Mathematically, this can be written as:

(2) $$\frac{d\sigma_{UTS}}{dX_{conc}}=C$$

By integrating on both sides of relation.

(3) $$\int d\sigma_{UTS}=\int C.dX_{con}$$

After integration the relationship becomes:

(4) $$\sigma_{UTS}=C_{1}(X_{conc})+C_{o}$$

where *C*_1_ and *C_o_* are integration constants which can further be written via *C*_1_ = *S_UTS_* and *C*_0_ = *σ_UTS_*_0_. This further implies that slope and intercept of this relation represents strength-sensitivity loss (S_UTS_) and approximated value/s of pristine specimen of PA-12 (σ_UTS0_), respectively. Hence, final form of the relation will become:

(5) $$\sigma_{UTS}=S_{UTS}(X_{conc})+\sigma_{UTS0}$$

Estimated values of *S_UTS_* and *σ_UTS0_* values were extracted via curve fitting procedure by ordinary least squares effectively. The units of S_UTS_ and σ_UTS0_ are MPa/wt% and MPa, respectively. The coefficient values of pristine and metallic fillers are presented in Table 1.

### 3.2. Mathematical Model for Elastic Modulus

Elastic modulus (*E*) was also modelled with changing *X_conc_*. However, in this case *E* was considered mathematically to vary with changing *X_conc_* as:

(6) $$\frac{dE}{dX_{conc}}=C+X_{Conc}$$

This is a kind of non-linear relationship based upon addition of a constant value to *X_conc_*. Now the relation is integrated on both sides.

(7) $$\int dE=\int (C+X_{Conc})dX_{Conc}$$

After integrating, the final form of the relation becomes:

(8) $$E=\frac{C_{2}}{2}{\left(X_{conc}\right)}^{2}+C_{1}\left(X_{conc}\right)+C_{o}$$

where *C*_2_, *C*_1_ and *C_o_* are replaced by *K_syn_*, *L_E_* and *E_o_* accordingly. Where *K_syn_*, *L_E_* and *E_o_* depict synergistic stiffening coefficient, elastic-modulus loss constant and Elastic modulus of pristine sample having units of MPa/(wt%)^2^, MPa/(wt%) and MPa, respectively.

(9) $$E={K_{syn}{\left(X_{conc}\right)}^{2}+L}_{E}(X_{conc})+E_{o}$$

The coefficients of elastic modulus versus metallic fillers are presented in Table 2.

## 4. Results and Discussions

### 4.1. Powder Characterization

#### 4.1.1. Particle Size Distribution Analysis

The particle size distribution of the PA12 matrix powder and metallic fillers (AlSi10Mg and 304 SS) was analyzed using a laser diffraction particle size analyzer (Malvern Mastersizer 3000, Malvern, UK). This analysis was performed to evaluate the size range and uniformity of the powders, as particle size significantly influences powder flowability, packing density, filler dispersion, and ultimately, the quality of injection-moulded parts.

The results revealed that the mean particle size (D50) for PA12 was 142.857 μm, indicating a relatively fine polymer powder suitable for homogeneous mixing and effective melting during injection moulding. The AlSi10Mg powder exhibited a significantly smaller mean particle size of 31.621 μm, which is advantageous for achieving a uniform dispersion and enhancing interfacial bonding with the PA12 matrix. In contrast, the 304 SS powder had a larger mean particle size of 244.962 μm, which can improve load transfer due to its higher contact area but may present challenges in achieving uniform distribution without adequate mixing energy.

**Figure 3:**
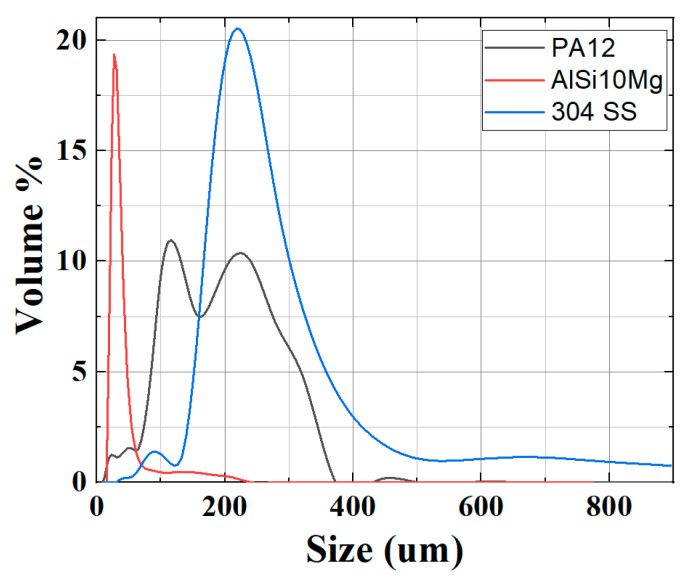
Particle size distribution curves of PA12, AlSi10Mg, and 304 SS powders showing distinct unimodal/bimodal profiles.

The particle size distribution curves (Figure 3) indicated that PA12 exhibited a bimodal distribution, suggesting the presence of both finer and coarser fractions, which may enhance packing density during moulding. AlSi10Mg displayed a narrow, unimodal distribution centered around its D50 value, while SS showed a broader distribution extending beyond 600 μm, reflecting its irregular morphology and larger particle sizes. Such variations in particle size and morphology between the fillers are expected to influence not only the mixing and compounding behavior but also the microstructural evolution and mechanical properties of the composites.

#### 4.1.2. Microstructure Analysis of Polymer and Metallic Particles

**Figure 4:**
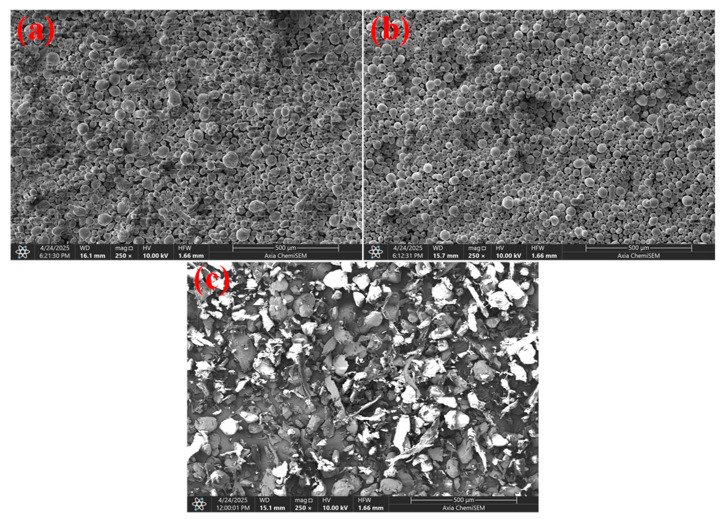
SEM micrographs of raw powders (**a**) AlSi10Mg, (**b**) 304 SS and (**c**) PA12.

SEM micrographs of the powders used in this study of AlSi10Mg particles, 304 SS particles, and PA12 polymer powder are shown in Figure 4. The SEM image reveals that the AlSi10Mg powder particles exhibit a predominantly spherical morphology with smooth surfaces, characteristic of gas-atomized metal powders. The spherical shape promotes excellent flowability and uniform dispersion within the polymer matrix during compounding. The relatively fine particle size distribution (mean size 31.6 μm) also enhances the surface area available for interfacial bonding with the PA12 matrix. The SS powder exhibits a spherical morphology with smooth and uniform surfaces, typical of SS powders produced for powder bed fusion or injection moulding applications. The average particle size (244.9 μm) falls within the optimal range for good packing density and consistent melting behavior during processing. The homogeneous size distribution and morphology support effective blending with polymers. The PA12 powder particles display an irregular morphology with sharp edges and faceted surfaces. This irregular shape is typical of atomized or mechanically processed polymer powders. The average size (142.9 μm) and angular morphology improve mechanical interlocking within the polymer but may present challenges in achieving a uniform dispersion. The SEM micrographs confirm distinct morphological differences between the metallic fillers and the polymer matrix powder. The spherical AlSi10Mg and SS powders facilitate flowability and uniform mixing, while the irregular PA12 particles provide mechanical anchoring sites within the matrix. These combined characteristics are expected to influence filler dispersion, interfacial bonding, and ultimately, the mechanical and tribological performance of the PA12-based composites. AlSi10Mg disperses better and yields stronger property gains than coarse 304 SS, which is consistent with polymer tribology composite reviews that stress interfacial area and clustering as primary drivers of performance [27].

**Figure 5:**
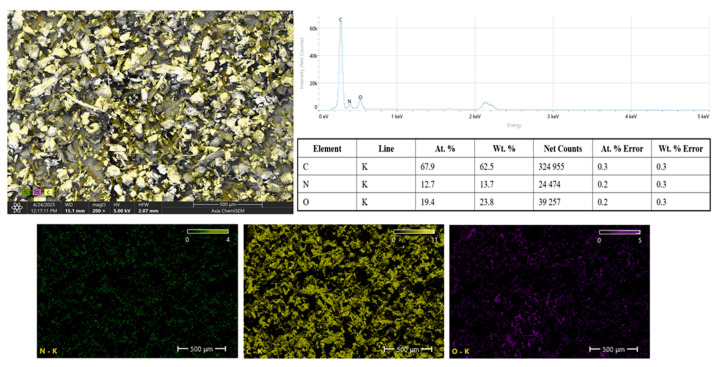
SEM–EDS analysis of PA12 powder confirming C, N, and O composition and uniform elemental distribution.

The EDS results of the PA12 powder are presented in Figure 5, comprising the SEM image with elemental overlays, the EDS spectrum, elemental composition data, and individual elemental mapping. The SEM micrograph shows a distribution of PA12 particles, and the overlaid elemental mapping highlights the presence of key elements characteristic of PA12: carbon (C), nitrogen (N), and oxygen (O). The EDS spectrum reveals a dominant peak for carbon, followed by smaller peaks corresponding to nitrogen and O. The quantitative EDS data confirm that PA12 powder primarily consists of carbon along with nitrogen which originates from the amide groups (-CONH-) in the polyamide structure, and oxygen, which is also associated with the amide linkage. The elemental mapping images further demonstrate a homogeneous distribution of C, N, and O across the PA12 particles, confirming the chemical uniformity of the powder. No foreign elements or contaminants were detected, indicating that the PA12 feedstock was pure and suitable for composite fabrication. The distinct and uniform elemental presence corroborates the expected stoichiometry of PA12, ensuring its compatibility for subsequent blending with metallic fillers during composite preparation.

**Figure 6:**
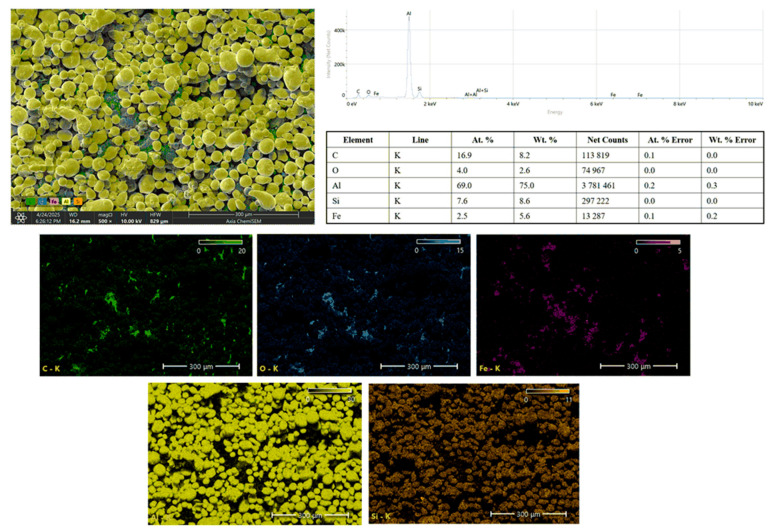
SEM–EDS spectra and elemental maps of AlSi10Mg powder showing Al, Si, Mg as dominant constituents with trace O and Fe.

The EDS results for the AlSi10Mg powder are presented in Figure 6. The EDS spectrum displays a dominant peak for aluminum (Al), confirming its major presence, accompanied by peaks for silicon (Si), magnesium (Mg, typically overlapped in Al peaks), trace iron (Fe), oxygen (O), and carbon (C). The quantitative analysis shows that the powder primarily consists of Al and Si, which are the principal constituents of the AlSi10Mg alloy. The presence of O is attributed to minor surface oxidation during storage and handling, while trace Fe could originate from contamination during atomization or handling equipment. C likely results from surface organic contaminants or adsorbed atmospheric hydrocarbons. The elemental mapping images demonstrate a homogeneous distribution of Al and Si throughout the powder particles, confirming the alloy’s uniformity. O mapping reveals localized surface oxidation areas, while carbon appears sporadically distributed, further indicating minor surface contamination. The trace iron is sparsely distributed, indicating minimal impurity levels that do not significantly affect the powder’s suitability for composite fabrication.

**Figure 7:**
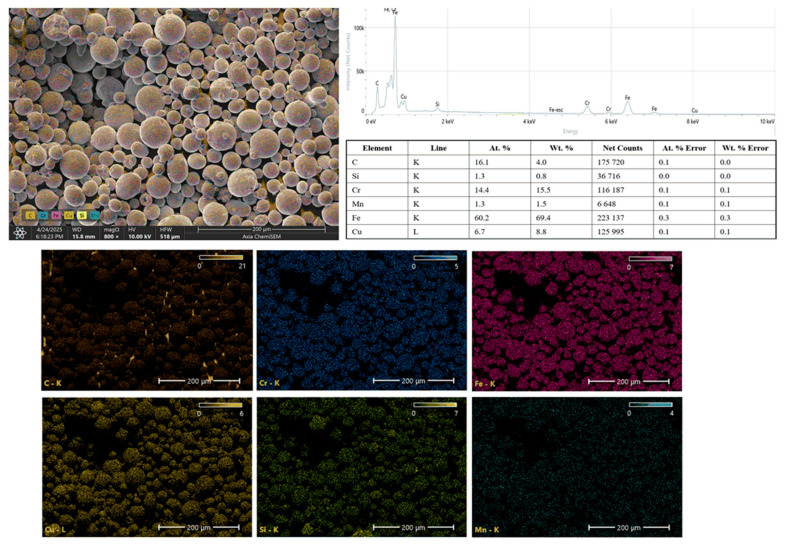
SEM–EDS spectra and elemental maps of 304 SS powder highlighting Fe, Cr, and minor alloying elements.

The EDS results of the 304 SS powder are shown in Figure 7. The EDS spectrum confirms the elemental composition characteristic of 304 SS, showing strong peaks for iron (Fe) and chromium (Cr), along with minor peaks for copper (Cu), carbon (C), silicon (Si), and manganese (Mn). The quantitative EDS results indicate that iron is the major constituent, contributing 69.4 wt.%, followed by Cr at 15.5 wt.%, which imparts corrosion resistance to the alloy. Cu is also detected, likely due to alloying or minor contamination during powder production, while Mn and Si are present in trace amounts, consistent with typical 304 SS composition. The presence of C can be attributed to surface contamination or carbon residues associated with powder processing and handling. The elemental mapping images illustrate a uniform distribution of Fe and Cr across all powder particles, demonstrating the homogeneity of the alloy. Copper, Mn, and Si are evenly dispersed in smaller quantities, confirming their roles as minor alloying elements. The presence of carbon is limited to isolated surface regions, suggesting minimal contamination.

### 4.2. Observation of Injection-Moulded Polymer and Composites

**Figure 8:**
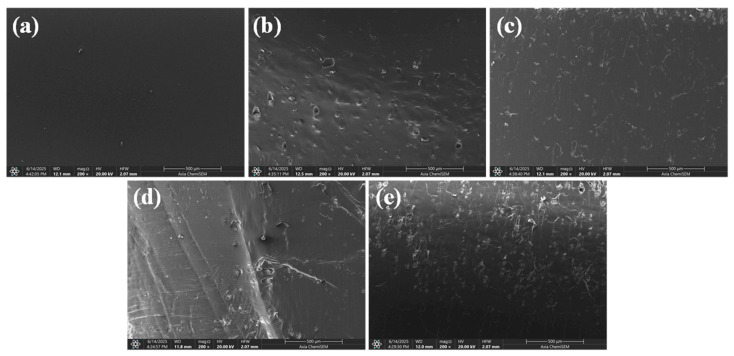
SEM micrographs of fracture surfaces: (**a**) neat PA12, (**b**) PA12 + 10% AlSi10Mg, (**c**) PA12 + 20% AlSi10Mg, (**d**) PA12 + 10% SS, and (**e**) PA12 + 20% SS.

The SEM image of neat PA12 (Figure 8a) displays a smooth and homogeneous surface morphology, indicative of a defect-free injection moulding process. The absence of visible voids or cracks suggests good polymer flow and solidification during moulding. The uniform texture reflects the isotropic nature of the polymer matrix, with no evidence of filler-related heterogeneity.

At 10% AlSi10Mg loading (Figure 8b), the surface shows localized dispersion of fine filler particles partially embedded within the PA12 matrix. Some small voids are observed, likely arising from incomplete wetting of metallic particles during moulding. Despite this, filler particles appear well-distributed, indicating good mixing and minimal agglomeration. The interfacial regions between the AlSi10Mg particles and the PA12 matrix appear intact, suggesting effective filler–matrix adhesion at this loading. At 20% AlSi10Mg (Figure 8c), the surface morphology reveals increased filler density with more visible particle outlines and minor agglomeration sites. While dispersion is largely maintained, the higher filler content introduces a slightly rougher surface texture with occasional micro-voids, possibly from trapped air or insufficient polymer wetting around clusters. The increased particle population is expected to improve stiffness and thermal stability but may also introduce brittleness if interfacial bonding is suboptimal.

The SEM micrograph for PA12 + 10% SS (Figure 8d) shows larger SS particles well-embedded in the matrix, with no significant detachment or interfacial gaps. The sharper nature of SS particles compared to AlSi10Mg creates pronounced filler–matrix interfaces. At 20% SS loading (Figure 8e), the micrograph depicts a dense filler distribution with increased surface roughness. The higher concentration of irregular SS particles leads to more prominent particle boundaries and localized microstructural heterogeneity. The polymer matrix shows evidence of strain marks and localized flow lines, which may result from increased viscosity during moulding at this filler level. Nevertheless, filler particles remain well-bonded, indicating that injection moulding parameters successfully mitigated interfacial defects even at higher reinforcement levels.

**Figure 9:**
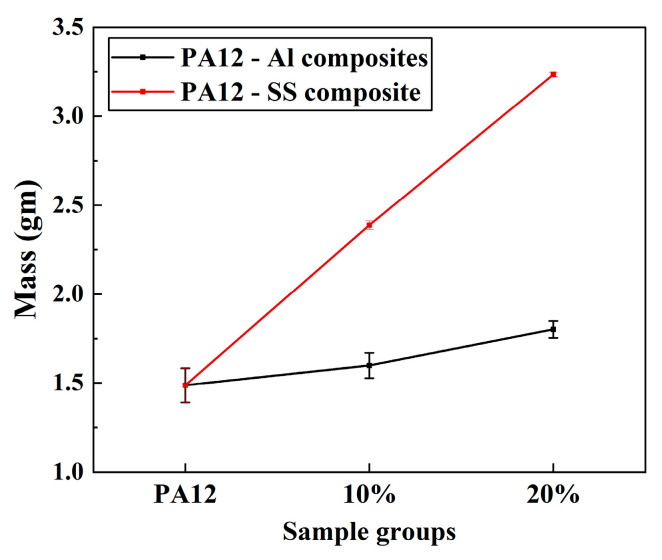
Measured mass of neat PA12 and composites, illustrating the impact of AlSi10Mg and SS fillers.

The mass measurements of neat PA12 and its composites reinforced with AlSi10Mg and 304 SS exhibit a clear correlation with the density and loading level of the metallic fillers. Neat PA12 recorded a mass of approximately 1.48 g, serving as the baseline reference. Upon reinforcement with AlSi10Mg, a moderate increase in mass was observed, with the 10% AlSi10Mg composite reaching 1.6 g and the 20% AlSi10Mg composite reaching 1.8 g (Figure 9). This gradual increase is attributed to the relatively low density of AlSi10Mg (2.68 g/cm^3^), which is only slightly higher than that of PA12 (1.02 g/cm^3^). Consequently, its incorporation primarily improves functional properties such as thermal stability and stiffness without imposing a substantial weight penalty, thereby maintaining the lightweight advantage of polymer-based composites. In contrast, the PA12-SS composites demonstrated a significantly larger increase in mass, which is directly related to the much higher density of 304 SS (7.9 g/cm^3^). The 10% SS composite reached 2.38 g, while the 20% SS composite increased to 3.2 g, more than doubling the mass relative to neat PA12. This pronounced rise results from the volumetric replacement of low-density PA12 with high-density SS, shifting the composite’s overall density upwards.

This aligns with the rule of mixtures for composite materials, where the theoretical density and resulting mass scale proportionally with the filler density and weight fraction. The minimal variation indicated by error bars confirms the consistency of injection moulding and uniform filler distribution, validating the fabrication methodology.

### 4.3. Surface Roughness

**Figure 10:**
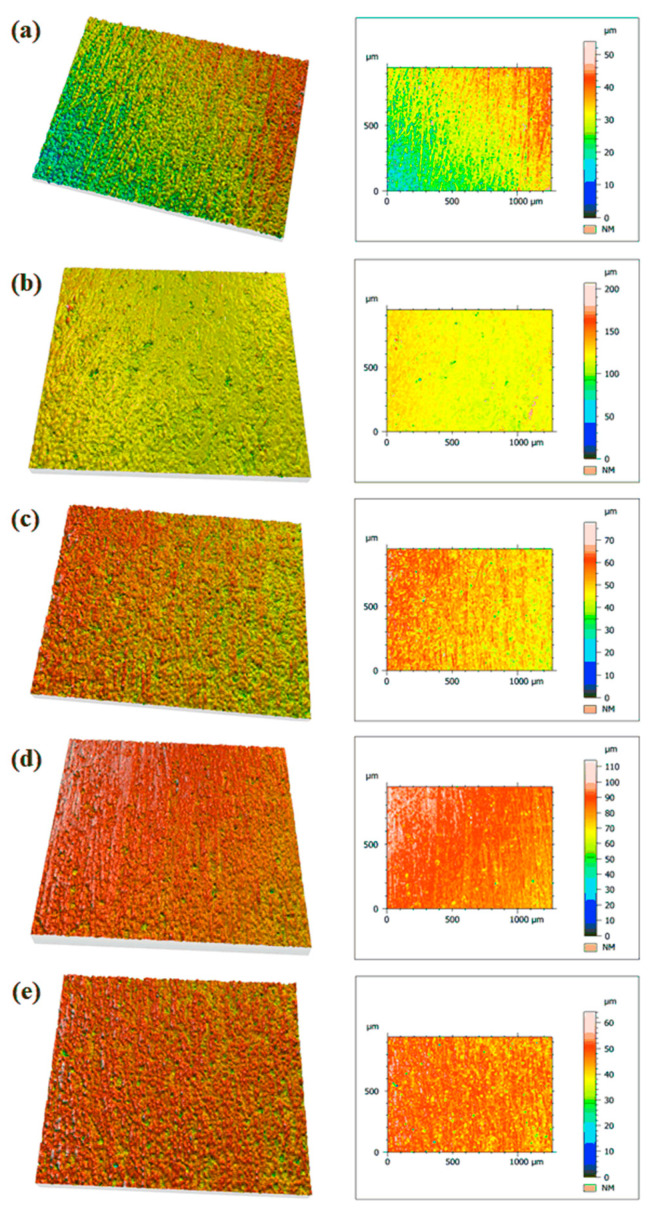
Mitutoyo Quick Vision Hyper 3D surface profilometry images of (**a**) neat PA12, (**b**) PA12 + 10% AlSi10Mg, (**c**) PA12 + 20% AlSi10Mg, (**d**) PA12 + 10% 304 SS, and (**e**) PA12 + 20% 304 SS.

The surface roughness (*Sa*) of neat PA12 and its composites reinforced with AlSi10Mg and 304 SS was evaluated using 3D optical profilometry, as shown in Figure 10. The corresponding *Sa* values are summarized in Table 3. The neat PA12 specimen exhibited a relatively smooth surface (*Sa* = 1.264 µm) with minor undulations arising from the injection moulding process, which is typical for unfilled thermoplastics. Upon addition of 10% AlSi10Mg, the surface roughness increased (*Sa* = 1.902 µm), indicating the influence of metallic filler particles protruding through the polymer matrix and creating localized peaks. However, at 20% AlSi10Mg, the surface roughness slightly decreased (*Sa* = 1.794 µm), suggesting improved packing and particle embedding due to higher filler loading and enhanced polymer–filler interaction, which mitigated surface irregularities despite the increased filler content.

In contrast, the SS-reinforced composites exhibited distinct roughness behavior. At 10% SS, the roughness remained close to neat PA12 (*Sa* = 1.297 µm), indicating uniform dispersion of the relatively larger SS particles within the matrix and effective encapsulation by PA12 during moulding. However, at 20% SS, the roughness increased substantially (*Sa* = 2.532 µm), which can be attributed to the larger particle size of SS fillers. Their irregular shapes likely disrupted the polymer surface during solidification, leading to higher asperity peaks and greater surface texturing.

The Mitutoyo Quick Vision Hyper 3D profilometry images corroborate these findings, where AlSi10Mg composites displayed a more uniformly textured surface, while SS composites exhibited rougher profiles with pronounced peaks and valleys, especially at higher filler loadings. Scientifically, this behavior is consistent with the influence of filler morphology and particle size: spherical AlSi10Mg particles integrate smoothly within the PA12 matrix, whereas irregular SS particles create surface protrusions and heterogeneity.

*Sa* is a critical parameter influencing the tribological and contact performance of polymer–metal composites. The observed trends suggest that AlSi10Mg fillers are more effective for applications requiring smoother surfaces and lower friction, while SS fillers may be beneficial where increased surface roughness improves mechanical interlocking or adhesion in secondary processes.

### 4.4. Tensile Properties

**Figure 11:**
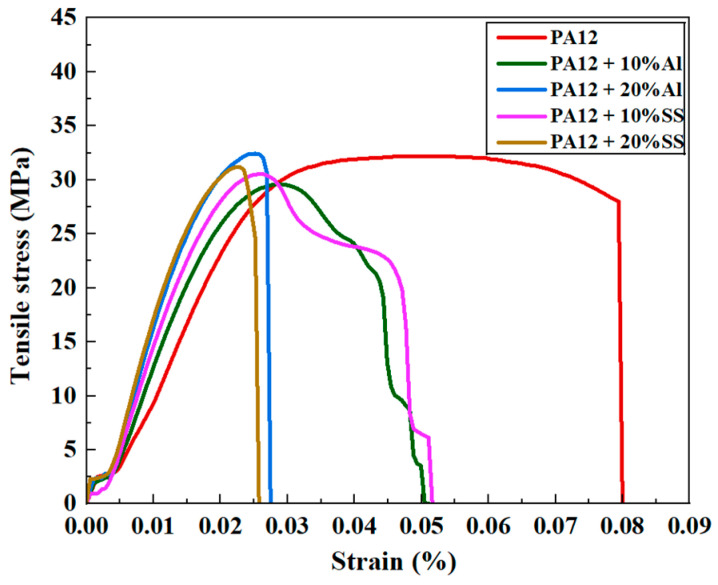
Stress–strain curves of neat PA12 and composites with 10 and 20 wt% AlSi10Mg or SS fillers.

The tensile stress–strain behavior of neat PA12 and its composites reinforced with AlSi10Mg and 304 SS at different filler loadings is shown in Figure 11. Neat PA12 demonstrated a typical ductile polymer response with a substantial elongation (8%), reflecting its inherent toughness and flexibility.

Upon the incorporation of AlSi10Mg, the composites displayed noticeable changes in tensile performance. At 10% AlSi10Mg, the tensile stress approximately reached 33 MPa, indicating effective stress transfer between the metallic filler and polymer matrix, likely due to good interfacial adhesion and uniform filler dispersion. However, elongation at break decreased significantly (3%), indicating a loss of ductility resulting from restricted polymer chain mobility caused by the rigid filler particles. For 20% AlSi10Mg, tensile strength decreased slightly compared to the 10% loading, while elongation further reduced. This reduction is likely attributed to particle agglomeration at higher loading levels, leading to localized stress concentrations and early fracture initiation.

The SS-filled composites followed a similar trend but exhibited slightly lower tensile strength than AlSi10Mg-filled samples. At 10% SS, tensile stress reached 31 MPa, comparable to neat PA12, but elongation reduced (2.5%), signifying a pronounced reduction in ductility. For 20% SS, tensile strength dropped further, accompanied by brittle fracture behavior, which is attributed to the larger particle size and irregular morphology of SS that can act as stress concentrators, hindering load transfer efficiency.

The results align with typical polymer–filler composite mechanics, where moderate filler loading improves strength via effective stress transfer, but excessive filler addition induces embrittlement due to poor polymer mobility and interfacial defects. Notably, AlSi10Mg reinforcement yielded better tensile strength improvements compared to SS, likely due to its spherical morphology, smaller particle size, and better dispersion within the matrix, which minimize stress concentration and improve filler–matrix bonding.

**Figure 12:**
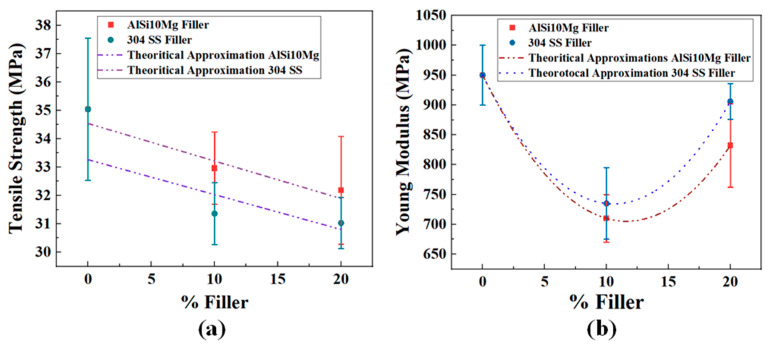
Properties obtained for PA12 after incorporating metallic fillers; (**a**) tensile strength and (**b**) Young’s modulus.

The tensile strength of neat PA12 was recorded as 35.04 MPa, reflecting the inherent ductility and toughness of the unreinforced polymer. Upon the incorporation of metallic fillers, a slight decrease in tensile strength was observed across all composite formulations. For the AlSi10Mg-reinforced composites, tensile strength values reduced to 32.96 MPa for 10% loading and further to 32.18 MPa for 20% loading. Similarly, the SS-reinforced composites exhibited tensile strengths of 31.36 MPa at 10% SS and 31.03 MPa at 20% SS (Figure 12a). This decline in tensile strength with filler addition can be attributed to two main factors. First, the introduction of rigid metallic particles restricts the ability of polymer chains to align and undergo plastic deformation under tensile loading, thereby reducing ductility and overall tensile performance. Second, fillers act as localized stress concentrators, particularly when there is incomplete wetting or weak adhesion at the polymer–filler interface. Stress concentration around these regions facilitates micro-crack initiation and accelerates fracture under tensile loading.

Interestingly, AlSi10Mg-reinforced composites maintained higher tensile strength than SS-filled counterparts at equivalent loadings. This difference can be explained by the smaller particle size (31.6 μm) of AlSi10Mg, which promotes better filler dispersion and reduces interfacial voids. In contrast, SS particles, with their larger average size (244.9 μm), are more prone to agglomeration and interfacial defects, thereby inducing premature fracture. These results align with the existing literature that correlates filler morphology and size to the efficiency of stress transfer in particulate-filled composites. The relatively small strength reduction across all samples also suggests that the injection moulding process was effective in achieving good filler distribution and wetting within the PA12 matrix.

The Young’s modulus of neat PA12 was measured as 0.95 GPa, which is typical of semi-crystalline polyamides with a balance of stiffness and flexibility. Following the incorporation of metallic fillers, the modulus decreased initially for both filler types at 10% loading, recording values of 0.71 GPa for AlSi10Mg and 0.73 GPa for SS (Figure 12b). This reduction at lower filler contents is counterintuitive but can be rationalized by considering the insufficient filler volume to form a continuous stress-bearing network, coupled with localized matrix softening around particle interfaces. The stress transfer efficiency from the polymer matrix to the rigid metallic fillers at these loadings is limited, and the discontinuous nature of the filler phase inhibits significant reinforcement effects.

At higher filler loading (20%), however, a partial recovery of stiffness was observed, with AlSi10Mg composites increasing to 0.83 GPa and SS composites reaching 0.90 GPa. This recovery indicates that at higher filler fractions, the metallic particles begin to interact sufficiently within the matrix to provide effective load transfer and reinforcement. Notably, SS composites achieved a slightly higher modulus than AlSi10Mg composites at 20% loading, owing to the inherently higher stiffness of SS relative to aluminum alloy. However, the irregular morphology of SS limits uniform stress transfer, preventing a more pronounced modulus gain.

The results suggest a dual-phase effect: at low filler loadings, isolated particles disrupt polymer chain mobility and reduce stiffness, while at higher filler loadings, particle-particle interactions and percolation effects enhance rigidity. The degree of this improvement is governed by filler type, morphology, and interfacial bonding. For AlSi10Mg, the smaller particle size and spherical geometry facilitate better interfacial adhesion and uniform stress distribution, while SS provides higher stiffness contributions but is prone to interfacial defects due to its irregularity and larger size.

The combined tensile strength and modulus data highlight the trade-off between stiffness and ductility in PA12–metal composites. While filler addition improves stiffness at higher loadings, it concurrently reduces ductility due to restricted polymer chain mobility and increased stress localization around filler particles. The superior retention of tensile strength in AlSi10Mg composites compared to SS suggests that particle morphology and size distribution significantly influence the balance of mechanical properties. AlSi10Mg particles promote more homogeneous stress transfer and fewer interfacial voids, while the larger, SS particles induce localized defects and early crack initiation despite their contribution to stiffness.

From an application standpoint, AlSi10Mg reinforcement is optimal for lightweight components requiring a balance of strength and stiffness, such as aerospace or automotive housings, whereas SS reinforcement is better suited for applications demanding rigidity and wear resistance, where weight and ductility are less critical. These findings align with classical composite mechanics, including the rule of mixtures and stress transfer theories, where filler geometry and interfacial bonding efficiency are decisive factors in determining mechanical behavior. Particle-filled PA12 typically shows modest stiffening/strengthening at low–moderate loadings; higher gains are reported with high-aspect-ratio or ceramic fillers (e.g., PA12–SiC, PA12–graphene) rather than spherical metals. Our trends (Al > SS) align with those reports, where better dispersion/surface area tracks higher E/σ improvements [28,29].

Table 4 illustrates the tensile strength and Young’s modulus of neat PA12 and composites with metallic fillers

### 4.5. Hardness

**Figure 13:**
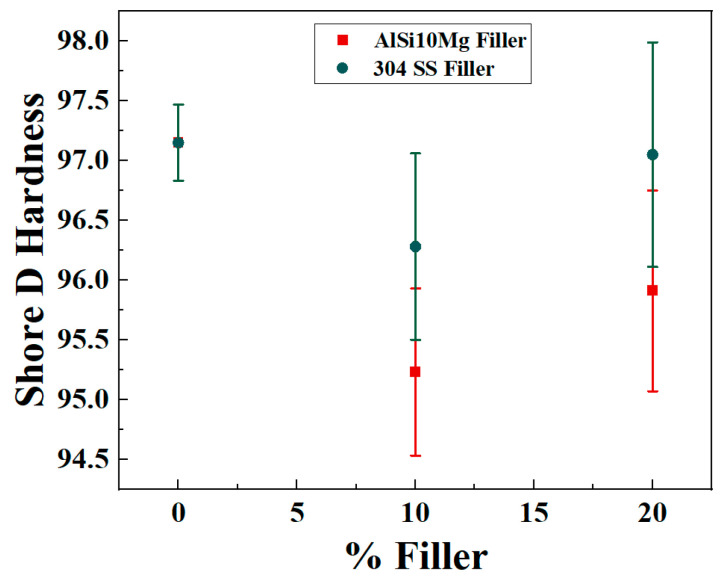
Shore D hardness of PA12 and composites at different filler loadings (mean ± SD).

The Shore D hardness results for neat PA12 and its composites reinforced with AlSi10Mg and 304 SS indicate minimal variation across the specimens, with all values remaining in a narrow range of 95–97 Shore D. Neat PA12 exhibited a hardness of 97.15, demonstrating its inherent rigidity as a semi-crystalline thermoplastic. The incorporation of metallic fillers resulted in only slight changes in hardness: PA12–10% Al (95.23), PA12–20% Al (95.91), PA12–10% SS (96.28), and PA12–20% SS (97.05). Figure 13 displays the Shore D hardness of PA12 and composites at different filler loadings.

The introduction of AlSi10Mg marginally reduced hardness compared to neat PA12. This minor decrease could be attributed to two factors: (i) the spherical morphology and finer particle size of AlSi10Mg result in more uniform dispersion without significantly altering the polymer matrix’s surface rigidity, and (ii) slight interfacial softening at the polymer–filler interface during processing may locally influence hardness values. Despite these changes, the variation remains negligible, indicating that AlSi10Mg fillers integrate well within PA12 without compromising its surface hardness.

SS-filled composites showed hardness values closer to that of neat PA12, with PA12–20% SS nearly matching the baseline hardness. The higher density of SS particles likely provide localized reinforcement beneath the indentation point during hardness testing, counterbalancing any polymer softening. This suggests that SS fillers effectively resist indentation due to their higher stiffness, thereby maintaining hardness levels comparable to neat PA12.

The limited change in hardness across all formulations suggests that Shore hardness is less sensitive to moderate filler loadings, particularly when fillers are well-dispersed and the polymer matrix dominates surface properties. Unlike tensile strength or modulus, hardness primarily reflects the surface’s immediate resistance to localized deformation rather than bulk stiffness or ductility. Since the metallic fillers are embedded within the polymer matrix and do not significantly protrude to the surface, their effect on hardness is less pronounced. Moreover, the high crystalline fraction of PA12 contributes strongly to its hardness, overshadowing minor filler-induced effects. The slight improvements with SS at higher loadings are likely due to localized constraint effects from the stiff metallic particles, while AlSi10Mg’s smoother morphology integrates more seamlessly, exerting minimal influence on surface rigidity.

The findings indicate that metallic reinforcement does not adversely affect PA12’s inherent hardness, ensuring that the composites retain surface resistance to indentation suitable for load-bearing and wear-prone applications. The ability to maintain hardness while tuning tensile and stiffness properties via filler selection provides design flexibility in tailoring PA12 composites for structural and functional uses. Table 5 illustrates the Shore D hardness values (mean ± standard deviation) of PA12 and composites at different filler loadings.

### 4.6. Tribological Performance

**Figure 14:**
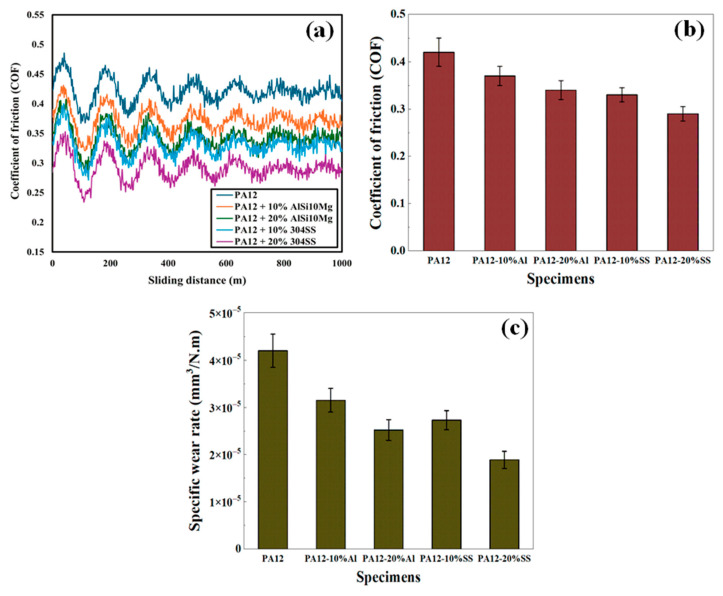
(**a**) Average COF versus sliding distance for PA12 and composites under 10 N load; (**b**) Average COF of PA12 and composites; (**c**) specific wear rate comparison of PA12 and composites.

The tribological response of neat PA12 and its composites was evaluated under dry sliding conditions using a ball-on-disc configuration. Figure 14 shows the evolution of the coefficient of friction (COF) with sliding distance, while Table 6 summarizes the average COF and specific wear rate values.

Neat PA12 exhibited a relatively high and unstable COF (0.42), typical of semicrystalline polymers where localized softening occurs at the sliding interface. Incorporation of metallic fillers reduced both the average COF and its fluctuation amplitude. At 10 wt% AlSi10Mg, the COF decreased to 0.37, and further to 0.34 at 20 wt%. SS fillers showed a stronger effect: 0.33 at 10 wt% and 0.29 at 20 wt%, with smoother friction curves over distance. The statistical analysis confirmed that these reductions were significant for all composites compared to neat PA12 (*p* < 0.05).

The specific wear rate of PA12 was measured at 4.2 × 10^−5^ mm^3^/N·m, dominated by adhesive wear and micro-ploughing. Both fillers improved wear resistance: AlSi10Mg composites showed a 25% reduction at 10 wt% and 40% at 20 wt%, while SS composites achieved a 35% and 55% reduction, respectively. The enhanced wear resistance of SS-filled composites is attributed to the higher hardness and load-bearing capacity of SS particles, which protect the matrix during sliding.

The tribological behaviour reflects the combined effects of filler morphology and dispersion. The fine AlSi10Mg particles reduce *Sa* and stabilize the interface, leading to moderate COF and wear reduction. The SS particles provide greater load support and COF reduction, but at the expense of higher abrasive wear at 20 wt%. Thus, AlSi10Mg is favourable where surface finish and smoother sliding are critical, while 304 SS offers superior wear protection for load-bearing applications. Hard, well-dispersed fillers tend to reduce wear and steady-state COF; as AlSi10Mg outperforms SS, paralleling PA12 studies where ceramic or solid-lubricant fills outperform baseline PA12 and where dispersion quality governs magnitude [28,30].

### 4.7. Differential Scanning Calorimetry (DSC)

**Figure 15:**
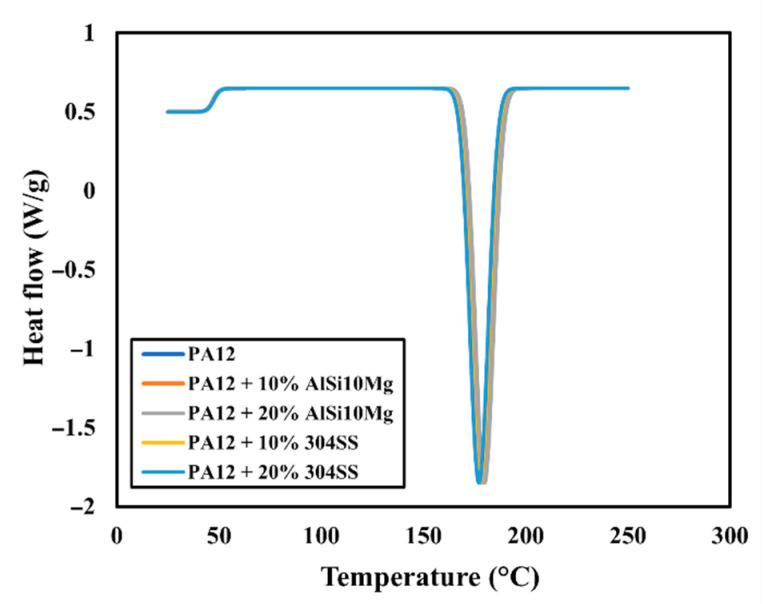
DSC thermograms (second heating) of neat PA12 and composites showing glass transition and melting peaks.

The DSC thermograms of neat PA12 and the composites are shown in Figure 15, with the thermal parameters summarized in Table 7. Neat PA12 exhibited a glass transition at 47 °C, in agreement with the literature values for semicrystalline PA12 [31]. Incorporation of metallic fillers had negligible effect on *Tg*, with only slight, statistically non-significant shifts (≤0.5 °C) observed for both AlSi10Mg and SS composites. This indicates that neither filler type strongly restricts the mobility of amorphous chain segments. The result is consistent with the weak interfacial interactions observed in SEM, where the fillers act as physical dispersoids rather than chemically bonded phases.

Neat PA12 displayed a sharp melting endotherm at 178 °C. AlSi10Mg-filled composites exhibited a slight increase in Tm (up to 180 °C at 20 wt%), whereas SS-filled composites showed a marginal decrease (177 °C at 20 wt%). The upward shift for AlSi10Mg suggests a nucleation-promoting effect of the fine spherical particles, leading to more ordered crystalline regions that require higher energy to melt. In contrast, the SS particles likely disrupt crystalline lamellae formation, producing less perfect crystals and lowering *Tm* slightly.

The degree of crystallinity (*Xc*) of neat PA12 was 31%. AlSi10Mg composites demonstrated an increase in Xc to 34% at 10 wt% and 34.5% at 20 wt%. This enhancement can be attributed to heterogeneous nucleation, where the smooth, high-surface-area particles act as nucleation sites during cooling, promoting more crystalline domains. By contrast, SS composites exhibited reduced crystallinity, decreasing to 28% at 20 wt%. This reduction arises from the particle morphology: large, irregular SS particles hinder chain mobility and interfere with spherulitic growth, producing incomplete or imperfect crystals.

PA12 systems with inorganic fillers commonly exhibit increased TGA onset/char and altered DSC transitions (e.g., glass-bead or mineral fills). The direction of improvement matches: thermally stable, inert fillers raise thermal stability without guaranteeing higher conductivity [32].

These findings corroborate the microstructural observations: AlSi10Mg composites showed homogeneous dispersion and good filler–matrix compatibility, while SS composites exhibited interfacial voids and clustering. From a performance perspective, higher crystallinity in AlSi10Mg composites supports improved stiffness and wear stability, whereas reduced crystallinity in SS composites aligns with their lower elongation at break and rougher fracture surfaces.

DSC results demonstrate that metallic fillers do not significantly alter *Tg* but exert a pronounced and opposite influence on crystallinity depending on particle morphology. This effect directly contributes to the microstructure–property correlations observed in the mechanical and tribological behaviour of the composites.

### 4.8. Thermogravimetric Analysis (TGA)

**Figure 16:**
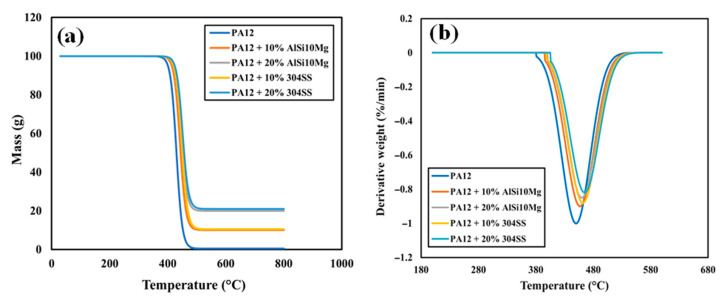
PA12 and composites; (**a**) TGA mass loss curves under nitrogen atmosphere; (**b**) DTG curves.

The TGA and derivative thermogravimetric (DTG) curves of neat PA12 and the composites under nitrogen atmosphere are shown in Figure 16a,b, with thermal parameters summarized in Table 8.

Neat PA12 showed a single-step thermal degradation process with an onset temperature (Tonset, 5% mass loss) of 405 °C and a maximum degradation rate (*Tmax*) of 450 °C. The decomposition pathway is typical of aliphatic polyamides, involving random scission of the polymer backbone and volatilization of degradation products [33]. The residual mass at 800 °C was negligible (<1%), consistent with complete thermal decomposition of the polymer matrix.

Incorporating AlSi10Mg fillers improved the thermal stability of PA12. *Tonset* increased by 12 °C at 10 wt% and 16 °C at 20 wt%, while *Tmax* shifted upward by 7–10 °C. These improvements can be attributed to a barrier effect: finely dispersed spherical particles reduce the diffusion of volatile degradation products, delaying the onset of mass loss. Furthermore, the higher thermal stability of AlSi10Mg facilitates heat dissipation, reducing localized overheating during decomposition. The residual mass at 800 °C increased in proportion to filler loading, reaching 20% at 20 wt%, corresponding to the metallic fraction.

The addition of SS resulted in an even more pronounced enhancement of thermal stability. *Tonset* increased by 18 °C at 10 wt% and 21 °C at 20 wt%, while *Tmax* shifted upward by 11–13 °C compared to neat PA12. The higher density and superior thermal stability of SS particles provide more effective heat shielding and delay the onset of thermal scission. At 800 °C, the residual mass corresponded closely to the metallic content (21% at 20 wt%), confirming the stability of SS particles under nitrogen.

When tested in air, all samples exhibited earlier degradation, with *Tonset* reduced by 40–50 °C relative to nitrogen. Nevertheless, composites retained their relative ranking: SS-filled composites were the most stable, followed by AlSi10Mg-filled composites, and neat PA12 was the least stable. The enhanced oxidative stability of the composites arises from the thermal shielding effect of metal fillers, which delay oxygen diffusion into the polymer matrix.

The thermal stability trends complement the DSC findings. AlSi10Mg promotes nucleation and crystallinity, while SS primarily enhances thermal endurance. Together, DSC and TGA analyses confirm that filler type not only modifies crystallization but also governs thermal degradation resistance, both of which are crucial for designing PA12 composites for high-temperature structural and tribological applications.

## 5. Conclusions

This work presented a comparative analysis of injection-moulded PA12 composites reinforced with AlSi10Mg and 304 SS fillers, highlighting the role of filler morphology in governing microstructure, thermal stability, and wear behaviour. Powder analysis confirmed that AlSi10Mg particles were fine and spherical (D50 ≈ 32 µm), whereas SS particles were coarse and angular (D50 ≈ 245 µm). These differences dictated the microstructural quality: AlSi10Mg composites exhibited homogeneous dispersion, while SS composites showed interfacial voids and clustering. Mechanical testing revealed that the elastic modulus of neat PA12 (1.2 GPa) increased by 20% with 20 wt% AlSi10Mg and by 28% with 20 wt% 304 SS. Tensile strength, however, decreased from 52 MPa for neat PA12 to 46 MPa (20 wt% AlSi10Mg) and 42 MPa (20 wt% SS), attributed to stress concentrations at particle agglomerates. Hardness remained nearly unchanged across all formulations (96–98 Shore D). Thermal analysis showed that AlSi10Mg slightly enhanced crystallinity (from 31% in neat PA12 to 34% at 20 wt%) and increased melting temperature by 2 °C, while SS reduced crystallinity to 28% but improved thermal stability, raising Tonset from 405 °C (PA12) to 426 °C (20 wt% 304 SS). Tribological testing demonstrated significant reductions in wear. The coefficient of friction decreased from 0.42 (PA12) to 0.34 (20 wt% AlSi10Mg) and 0.29 (20 wt% 304 SS), while wear rates were reduced by 40% and 55%, respectively. SEM of worn surfaces revealed smoother tracks for AlSi10Mg composites and abrasive grooves for SS composites. The AlSi10Mg fillers promote improved crystallinity, smoother surfaces, and moderate wear resistance, making them suitable for applications requiring dimensional stability and low surface roughness. 304 SS fillers, though detrimental to crystallinity, offer superior stiffness, wear resistance, and thermal stability, making them advantageous in high-load and high-temperature environments. The ranking for the fillers performance resulted as microstructure (dispersion/porosity)—AlSi10Mg > 304SS, mechanical properties (ΔE, ΔσUTS)—AlSi10Mg > 304SS, thermal stability (TGA onset/residue)—304SS > AlSi10Mg and wear behaviour (COF, wear rate)—AlSi10Mg > 304SS.

## Figures and Tables

**Table 1:** Coefficient values for estimation of σ_UTS_ w.r.t Filler.

Filler	Baseline UTS, σ (MPa)	UTS Loss Rate (MPa/wt%)
AlSi10Mg	34.539 ± 0.633	−0.1233 ± 0.0495
304 SS	33.261 ± 1.618	−0.1230 ± 0.1040

**Table 2:** Coefficient values for estimation of elastic modulus w.r.t filler.

Filler	E_0_ (MPa)	L_E_ (Elastic-Modulus Loss Constant) [MPa/wt%]	K_Syn (Synergistic Stiffening Coefficient) [MPa/(wt%)^2^]	Xcon (Critical % at Minimum E)
AlSi10Mg	950	−42.1	1.81	≈11.6
304 SS	950	−40.8	1.93	≈10.6

**Table 3:** Surface roughness (Sa) values of neat PA12 and composites reinforced with 10 and 20 wt% AlSi10Mg or 304 SS.

Specimen	Surface Roughness, Sa (µm)
PA12	1.264
PA12 + 10% AlSi10Mg	1.902
PA12 + 20% AlSi10Mg	1.794
PA12 + 10% 304 SS	1.297
PA12 + 20% 304 SS	2.532

**Table 4:** Tensile strength and Young’s modulus of neat PA12 and composites with metallic fillers.

	Tensile Properties	
Specimen	Tensile Strength (MPa)	Young’s Modulus (GPa)
PA12	35.04 + 2.51	0.95 + 0.05
PA12 + 10% AlSi10Mg	32.96 + 1.27	0.71 + 0.04
PA12 + 20% AlSi10Mg	32.18 + 1.90	0.83 + 0.07
PA12 + 10% 304 SS	31.36 + 0.91	0.73 + 0.06
PA12 + 20% 304 SS	31.03 + 0.78	0.90 + 0.03

**Table 5:** Shore D hardness values (mean ± standard deviation) of PA12 and composites at different filler loadings.

Specimen	Hardness
PA12	97.15 + 0.32
PA12 + 10% AlSi10Mg	95.23 + 0.70
PA12 + 20% AlSi10Mg	95.91 + 0.84
PA12 + 10% 304 SS	96.28 + 0.78
PA12 + 20% 304 SS	97.05 + 0.94

**Table 6:** Tribological results of neat PA12 and composites: average COF and specific wear rate.

Specimen	Avg COF	Wear Rate (mm^3^/N.m)
PA12	0.42	4.2 × 10^−5^
PA12 + 10% AlSi10Mg	0.37	3.15 × 10^−5^
PA12 + 20% AlSi10Mg	0.34	2.52 × 10^−5^
PA12 + 10% 304 SS	0.33	2.73 × 10^−5^
PA12 + 20% 304 SS	0.29	1.89 × 10^−5^

**Table 7:** DSC thermal parameters of PA12 and composites: glass transition temperature (Tg), melting temperature (Tm), and degree of crystallinity (Xc).

Specimen	Tg (°C)	Tm (°C)	Xc (%)
PA12	47	178	31
PA12 + 10% AlSi10Mg	47.2	179.2	34
PA12 + 20% AlSi10Mg	47.5	179.8	34.5
PA12 + 10% 304 SS	47.1	177.6	29.5
PA12 + 20% 304 SS	47.3	177.2	28

**Table 8:** TGA results of PA12 and composites: onset degradation temperature (Tonset) and peak degradation temperature (Tmax).

Specimen	Tonset (°C)	Tmax (°C)
PA12	405	450
PA12 + 10% AlSi10Mg	417	457
PA12 + 20% AlSi10Mg	421	460
PA12 + 10% 304 SS	423	461
PA12 + 20% 304 SS	426	463

## Data Availability

The original contributions presented in the study are included in the article, further inquiries can be directed to the corresponding authors.
